# Survival in infants treated with sebelipase Alfa for lysosomal acid lipase deficiency: an open-label, multicenter, dose-escalation study

**DOI:** 10.1186/s13023-017-0587-3

**Published:** 2017-02-08

**Authors:** Simon A. Jones, Sandra Rojas-Caro, Anthony G. Quinn, Mark Friedman, Sachin Marulkar, Fatih Ezgu, Osama Zaki, J. Jay Gargus, Joanne Hughes, Dominique Plantaz, Roshni Vara, Stephen Eckert, Jean-Baptiste Arnoux, Anais Brassier, Kim-Hanh Le Quan Sang, Vassili Valayannopoulos

**Affiliations:** 1 Manchester Centre for Genomic Medicine, 6th floor, St Mary’s Hospital, Central Manchester Foundation Trust, University of Manchester, Oxford Road, Manchester, M13 9WL UK; 2 Synageva BioPharma Corp., 33 Hayden Avenue, Lexington, MA 02421 USA; 3 Alexion Pharmaceuticals, Inc., 100 College Street, New Haven, CT 06510 USA; 4 Gazi University Faculty of Medicine, Gazi Hospital, 10th Floor, Beşevler Ankara, Turkey; 5 Ain Shams University Pediatrics Hospital, 3, Kamal Raslan, Heliopolis, Cairo, 11771 Egypt; 6 University of California, Irvine, 2056 Hewitt Hall, 843 Health Sciences Road, Irvine, CA 92697 USA; 7 Temple Street Children’s University Hospital, 1 Temple Street, Dublin, 1 Ireland; 8 Hôpital Couple-Enfant CHU Grenoble, Avenue Maquis du Grésivaudan, 38700 La Tronche, Grenoble, France; 9 Evelina Children’s Hospital, Westminster Bridge Road, London, SE1 7EH UK; 10 Hôpital Necker-Enfants Malades and IMAGINE Institute, 149 Rue de Sèvres, 75015 Paris, France; 11 Present: IDBioPharm Consulting, LLC, Boston, MA USA; 12 Present: Sanofi Genzyme, Cambridge, MA USA

**Keywords:** Lysosomal acid lipase deficiency, Wolman disease, Sebelipase alfa, Survival, Hemophagocytic lymphohistiocytosis

## Abstract

**Background:**

Infants presenting with lysosomal acid lipase deficiency have marked failure to thrive, diarrhea, massive hepatosplenomegaly, anemia, rapidly progressive liver disease, and death typically in the first 6 months of life; the only available potential treatment has been hematopoietic stem cell transplantation, which is associated with high morbidity and mortality in this population. The study objective was to evaluate safety and efficacy (including survival) of enzyme replacement with sebelipase alfa in infants with lysosomal acid lipase deficiency. This is an ongoing multicenter, open-label, phase 2/3 study conducted in nine countries. The study enrolled infants with growth failure prior to 6 months of age with rapidly progressive lysosomal acid lipase deficiency; they received once-weekly doses of sebelipase alfa initiated at 0.35 mg/kg with intrapatient dose escalation up to 5 mg/kg. The main outcome of interest is survival to 12 months and survival beyond 24 months of age.

**Results:**

Nine patients were enrolled; median age at baseline was 3.0 months (range 1.1–5.8 months). Sixty-seven percent (exact 95% CI 30%–93%) of sebelipase alfa–treated infants survived to 12 months of age compared with 0% (exact 95% CI 0%–16%) for a historical control group (*n* = 21). Patients who survived to age 12 months exhibited improvements in weight-for-age, reductions in markers of liver dysfunction and hepatosplenomegaly, and improvements in anemia and gastrointestinal symptoms. Three deaths occurred early (first few months of life), two patients died because of advanced disease, and a third patient died following complications of non-protocol-specified abdominal paracentesis. A fourth death occurred at 15 months of age and was related to other clinical conditions. The five surviving patients have survived to age ≥24 months with continued sebelipase alfa treatment; all have displayed marked improvement in growth parameters and liver function. Serious adverse events considered related to sebelipase alfa were reported in one of the nine infants (infusion reaction: tachycardia, pallor, chills, and pyrexia). Most infusion-associated reactions were mild and non-serious.

**Conclusion:**

Sebelipase alfa markedly improved survival with substantial clinically meaningful improvements in growth and other key disease manifestations in infants with rapidly progressive lysosomal acid lipase deficiency

**Trial registration:**

Clinicaltrials.gov NCT01371825. Registered 9 June 2011.

**Electronic supplementary material:**

The online version of this article (doi:10.1186/s13023-017-0587-3) contains supplementary material, which is available to authorized users.

## Background

Lysosomal acid lipase (LAL) deficiency is an autosomal-recessive disorder due to deficiency of LAL, the enzyme that hydrolyzes cholesteryl esters and triglycerides (Online Mendelian Inheritance in Man® database #278000) [1]. The enzyme deficiency is due to *LIPA* mutations and leads to lysosomal lipid accumulation in many types of cells and tissues [2, 3]. Like other lysosomal storage diseases, this disease presents across an age continuum from infancy to adulthood [4]. The most rapidly progressive presentation of LAL deficiency occurs in infants (in infants, LAL deficiency has historically been known as Wolman disease [5, 6]), and usually leads to death before 6 months of age [2, 7]. Affected infants show evidence of the disease within the first few months of life, with diarrhea, marked failure to thrive, abdominal distension, massive hepatosplenomegaly, anemia, and rapidly progressive liver dysfunction. Affected children and adults demonstrate a number of abnormalities similar to those seen in infants, including hepatomegaly, elevated serum transaminase levels, liver fibrosis, and dyslipidemia with progression to cirrhosis, and other complications related to end-stage liver disease [2, 4, 8, 9]. Enzyme replacement with sebelipase alfa in children and adults has recently been shown to produce significant improvements in a broad range of disease-related manifestations [10].

Although the disease presentation in infants is striking, familiarity with the disease is low and there are a number of published cases where the correct diagnosis was not made until after death [11]. In untreated infants the disease progresses rapidly, with death occurring within a few weeks after diagnosis (median age at death, 3.7 months) [7]. Prior to the availability of sebelipase alfa, treatment options centered upon supportive interventions, including blood transfusions and nutritional supplementation. Hematopoietic stem cell transplantation (HSCT) and liver transplantation have been used in a small number of cases but outcomes remain very poor, with few reported survivors of HSCT [12–17]. In case reports involving a total of 12 patients with early-onset LAL deficiency who received HSCT, nine patients died within 8 months post-transplant and one died approximately 6 years after receiving HSCT; most of these deaths resulted from either progression of LAL deficiency or HSCT-related complications such as infection, graft-versus-host disease, and/or graft failure.

Enzyme replacement therapy (ERT) has had a dramatic impact in a number of lysosomal storage disorders including Gaucher disease, Fabry disease, Pompe disease, and the mucopolysaccharidoses. Sebelipase alfa (Alexion Pharmaceuticals, Inc., New Haven, Connecticut, USA) is a recombinant human LAL developed as an ERT for LAL deficiency [18]. Here we report survival to 12 months of age (the primary endpoint), survival to 24 months of age, safety, and disease-related outcomes in infants with rapidly progressive LAL deficiency who received sebelipase alfa.

## Methods

### Study design and patients

This is an ongoing, global, phase 2/3, open-label, repeat-dose, intrapatient dose-escalation study of the efficacy and safety of sebelipase alfa in infants (VITAL, Survival of LAL-D Infants Treated With Sebelipase Alfa; NCT01371825) [19]. Twelve centers in nine countries are participating (Additional file 1, eStudy centers), with initiation on April 11, 2011. Patients were enrolled over a 2.5-year period and the data cut-off date for analysis of survival to 12 months of age (primary endpoint) was June 10, 2014. All surviving patients continue to receive sebelipase alfa treatment in an ongoing extension study; the cut-off date for analysis of survival to 24 months of age was January 26, 2016. Patients received sebelipase alfa, with treatment initiated at 0.35 mg/kg once weekly and escalation up to 3 mg/kg based on clinical response (Additional file 1, eDose adjustment based on clinical response). A further dose escalation to 5 mg/kg once weekly was allowed upon consultation between the investigator and study sponsor, including instances where there was insufficient clinical response in patients with potentially neutralizing anti-drug antibodies (ADAs; Additional file 1, eMeasurements). Reduced dosing frequency to once every other week was allowed, based on clinical response and investigator discretion in consultation with the sponsor. The rapid disease progression and high mortality rate in patients with LAL deficiency precluded the use of a placebo control group in this study; a population of untreated LAL-deficient patients with growth failure from a natural history study served as a historical control group (data on file, Alexion Pharmaceuticals, Inc.) [7].

Eligible patients were under 8 months of age as of the date of the anticipated first infusion, had a diagnosis of LAL deficiency confirmed (with LAL enzyme activity testing and/or *LIPA* mutation analysis), and demonstrated growth failure or other evidence of rapidly progressive disease with onset before 6 months of age (Additional file 1, eEvidence of rapidly progressive disease as inclusion criterion). Growth failure criteria were weight decreasing across two or more of the 11 major centiles (99^th^, 97^th^, 95^th^, 90^th^, 75^th^, 50^th^, 25^th^, 10^th^, 5^th^, 3^rd^, 1^st^) on a standard World Health Organization (WHO) weight-for-age (WFA) chart, or body weight below the 10^th^ centile on a standard WHO WFA chart and no weight gain during the 2 weeks before screening, or loss of greater than 5% of birth weight after 2 weeks of age. Key exclusion criteria included myeloablative preparation or other transplant conditioning and previous HSCT or liver transplant.

The primary objective was to evaluate the effect of sebelipase alfa therapy on survival at 12 months of age in LAL-deficient infants with growth failure. Secondary objectives included evaluation of safety, survival at 6-month intervals after 12 months of age, changes in centiles for WFA and length-for-age (LFA) [20], growth status indicators of underweight, wasting, and stunting [21], markers of liver injury (alanine aminotransferase [ALT], aspartate aminotransferase [AST]), liver function (total bilirubin, γ-glutamyltransferase [GGT], and alkaline phosphatase), transfusion-free hemoglobin normalization (TFHN; Additional file 1 [eDefinition of transfusion-free hemoglobin normalization] for description), and changes in liver and spleen size and volume, based on physical examination and ultrasound at scheduled time points (physical examination: weeks 3, 9, 15, 20, and 24, and every 3 months thereafter; abdominal ultrasound: weeks 0, 12, 24, and annually thereafter). Adverse events data are being collected and analyzed on an ongoing basis. Exploratory objectives included the effects of sebelipase alfa on serum lipid levels (low-density lipoprotein cholesterol, triglycerides, high-density lipoprotein cholesterol, and total cholesterol). The Denver II Developmental Screening Test [22] for evaluating fine motor–adaptive, gross motor, language, and personal-social skills (performance-based and parent-reported) was also included. Results for each of the four assessment categories of the test were interpreted by the evaluator as “normal”, “suspect”, or “untestable”.

Safety was assessed by ascertainment of adverse events, discontinuations, deaths, infusion-associated reactions (IARs; Additional file 1, eDefinition of infusion-associated reaction), ADAs, clinical laboratory tests, vital signs, physical examinations, and electrocardiograms.

### Statistical analysis

The primary efficacy set was defined as patients in the full analysis set who were 8 months of age or younger on the date of their first infusion (*n* = 9). Survival was compared to survival in a historical cohort of untreated infants with confirmed diagnosis of LAL deficiency, who had similar clinical characteristics (data on file, Alexion Pharmaceuticals, Inc.) [7]. All hematological, liver biochemical, and lipid analyses were performed by local laboratories, and assessed using age- and gender-specific normal values at the time of the test. Details of planned statistical methods including sample size are described in Additional file 1 (eStatistical methods and determination of sample size).

## Results

### Patient characteristics and treatment

**Fig. 1:**
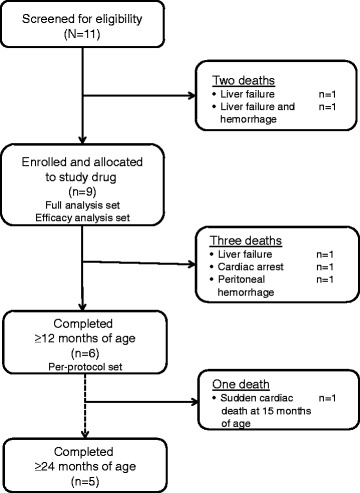
Patient flow and disposition. Two patients died during screening; three patients died after enrollment, all of whom received four or fewer doses of sebelipase alfa

Nine patients were enrolled and received weekly infusions of sebelipase alfa (Fig. 1). The median age of patients at the time of initiation of treatment was 3.0 months (range 1.1–5.8 months); eight of the nine patients met the pre-specified growth failure criteria. In seven of the nine patients, sebelipase alfa doses were escalated to once-weekly doses of 1 mg/kg, 3 mg/kg, or 5 mg/kg. One patient with neutralizing antibodies and less-than-expected improvements in growth compared with other infants received a dose increase to 5 mg/kg. A second patient had doses escalated to 5 mg/kg weekly due to persistence of elevated transaminase levels, hypoalbuminemia, poor weight gain, and poor general well-being. At the primary analysis data cut-off date, patients had received a total of 462 infusions (including 141 infusions at 1.0 mg/kg, 295 infusions at 3.0 mg/kg, and 8 infusions at 5.0 mg/kg). Median duration of exposure was 60.3 weeks (range 0.1–164.7 weeks). At the follow-up cut-off date (January 26, 2016) for the five patients surviving to age 24 months, a total of 838 sebelipase alfa infusions had been administered. All five ongoing patients had required a dose escalation to 3.0 mg/kg and two patients were receiving 5.0 mg/kg. The protocol contains clear criteria for dose escalation (Additional file 1, eDose adjustment based on clinical response), first to 3 mg/kg and then to 5 mg/kg. As well as these criteria, other factors also contributed to decisions to escalate dosing, including WFA, hepatomegaly, lymphadenopathy, and presence of anti-drug, anti-enzyme, or anti-uptake antibodies. The current clinical status, documented presence of ADAs, and reasons for dose escalations for each of the five ongoing patients are summarized in Additional file 1: Table S1.

*LIPA* mutation data were available for six patients (Additional file 1: Table S2, LAL enzyme activity and *LIPA* genotypes). None of them had a copy of the previously described c.894 G > A common exon 8 splice junction mutation, which is commonly found in LAL deficiency presenting in children and adults [23, 24]. Two patients were homozygous and one patient heterozygous for documented causative mutations. The three other patients with genotyping data were either homozygous (one patient) or heterozygous (two patients) for undocumented mutations. In the three patients without confirmed pathological mutations, LAL deficiency diagnosis was confirmed based on LAL enzyme activity testing (Additional file 1: Table S2, LAL enzyme activity and *LIPA* genotypes).

In addition to growth failure, diarrhea, vomiting, abdominal distension, and marked hepatosplenomegaly at baseline, patients had anemia (*n* = 6), adrenal calcification (*n* = 9), ascites (*n* = 4), and/or thrombocytopenia (*n* = 3) (Table 1). The baseline liver parameters revealed significant underlying liver injury/dysfunction with a median ALT level of 145 U/L (2.42 μkat/L) (range 16.0–297.0 U/L [0.27–4.96 μkat/L]) and median AST level of 125 U/L (2.09 μkat/L) (range 71.0–716.0 U/L [1.19–11.96 μkat/L]), and abnormally high GGT and total bilirubin levels in four and three patients, respectively (Additional file 1: Table S3). Although baseline high-density lipoprotein cholesterol and triglyceride levels were abnormal in most patients, no consistent pattern of abnormality was seen in low-density lipoprotein cholesterol levels (data not shown). Supportive treatments during the screening period included a low-fat/low-cholesterol diet, nutritional supplements including medium-chain triglyceride formula, parenteral nutrition, administration of bile acid chelators such as cholestyramine, and transfusions.

**Table 1:** Patient demographics and baseline disease characteristics (full analysis set)

Parameter	Patients (*N* = 9)
Age at treatment initiation, months	
Median (range)	3.0 (1.1–5.8)
Gender, *n* (%)	
Male	5 (56)
Female	4 (44)
Race	
White	4 (44)
Black	1 (11)
Asian	1 (11)
Unknown^*^	3 (33)
Age at symptom onset, months	
Range	0–5.0
Age at diagnosis, months	
Range	0–5.8
LAL deficiency manifestations, *n* (%)	
Hepatosplenomegaly	9 (100)
Abdominal distension	9 (100)
Vomiting	9 (100)
Diarrhea	9 (100)
Adrenal calcifications	9 (100)
Failure to thrive	9 (100)
Anemia	6 (67)
Ascites	4 (44)
Thrombocytopenia (<150 × 10^9^/L)	3 (33)
Hematological parameters, median (range)	
Hemoglobin, g/L	93 (1.4–103.0)
Platelets, 10^9^/L	173 (2.6–563)
Serum ferritin, μg/L, median (range)	586 (253–48,740)
Multiple organ dysfunction syndrome, *n* (%)	3 (33)
Growth failure/entry criteria met,^†^ *n* (%)	
Weight decreasing across ≥2 of the 11 major centiles	7 (78)
Body weight <10^th^ centile and no weight increase during 2 weeks before screening	1 (11)
Loss of >5% of birth weight after 2 weeks of age	0
Rapidly progressive course of LAL deficiency without meeting growth failure criteria	1 (11)

All hematological analyses were performed by local laboratories; assessment of normal/abnormal results was based on the age- and gender-specific normal range provided by the local laboratory at the time of the test

^*^Race was not reported for three patients enrolled and treated in France in compliance with that country’s regulations

^†^Patients were required to meet at least one of these criteria

### Survival

**Fig. 2:**
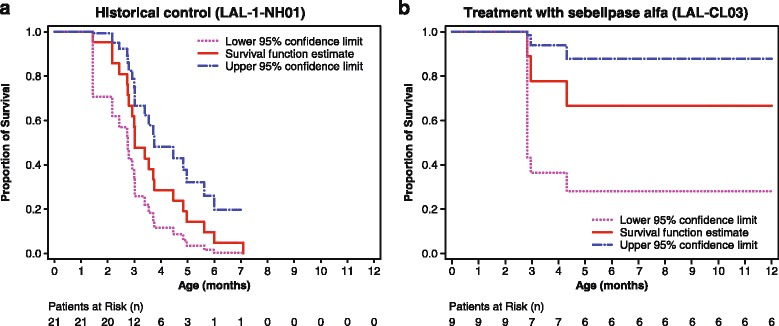
Survival from birth to 12 months of age. Kaplan-Meier analyses of (**a**) historical control group of untreated patients with early growth failure (*n* = 21) from natural history study of infants with LAL deficiency (LAL-1-NH01; data on file, Alexion Pharmaceuticals, Inc.) [7] and (**b**) the primary efficacy set of the present phase 2/3 study (*N* = 9). Patients in the historical control group were considered untreated if they had not received hematopoietic stem cell transplant, liver transplant, or enzyme replacement therapy. Growth failure was defined by 1) decreased body weight across ≥2 of the 11 major centiles on a standard WHO weight-for-age chart, or 2) body weight in kilograms below the 10^th^ centile on a standard WHO weight-for-age chart and no weight gain for the previous 2 weeks, or 3) loss of ≥5% of birth weight in children who are >2 weeks of age

The primary endpoint of survival to 12 months of age was met by six of the nine patients (67%; exact 95% CI 29.9–92.5) and all six patients were alive as of June 10, 2014 (ages 12.0, 15.7, 15.8, 20.4, 25.1, and 42.2 months). Of the three patients who died before age 12 months, two received only one infusion and one received only four infusions of sebelipase alfa; these deaths were deemed not related or unlikely to be related to the drug. The median age at death for the three deceased patients was 2.9 months (range 2.8–4.3 months). One of the six surviving infants at 12 months died at 15 months of age. For this patient, the stated cause of death based on autopsy findings was sudden cardiac death, which was assessed as unlikely to be related to sebelipase alfa. This infant manifested other comorbidities not typically associated with LAL deficiency including arterial hypertension, which preceded the diagnosis of LAL deficiency, a patent foramen ovale, and hemoglobin E disease. The hypertension, which existed prior to enrollment in the study, was thought to be associated with fluid overload and was being treated with furosemide, amlodipine, and clonidine. Elevated levels of renin and aldosterone were observed; however, renal size (measured by ultrasound) was within the upper limit of the normal range. Post-mortem findings included evidence of early cirrhosis in the liver. The lungs and intestine showed xanthomatous changes; also spleen and bone marrow showed widespread lipid vacuoles and lipid-laden macrophages, consistent with underlying disease. Results of Kaplan-Meier survival analyses are shown in Fig. 2 for the patients receiving sebelipase alfa and for untreated patients with growth failure in the historical control group (data on file, Alexion Pharmaceuticals, Inc.) [7]. Survival at age 12 months in the historical control group (*n* = 21) was 0% (exact 95% CI 0–16.1).

The remaining five patients have survived to ≥24 months of age (median 3 years, 4 months [range 2 years, 11 months to 5 years, 2 months]) as of the January 26, 2016 extension analysis cut-off date, and all have continued to receive sebelipase alfa treatment; survivors’ median time in the study at the cut-off date was 2 years, 10 months (maximum duration of exposure to study drug was 4 years, 9 months).

### Effects on growth and other important disease-related abnormalities

**Fig. 3:**
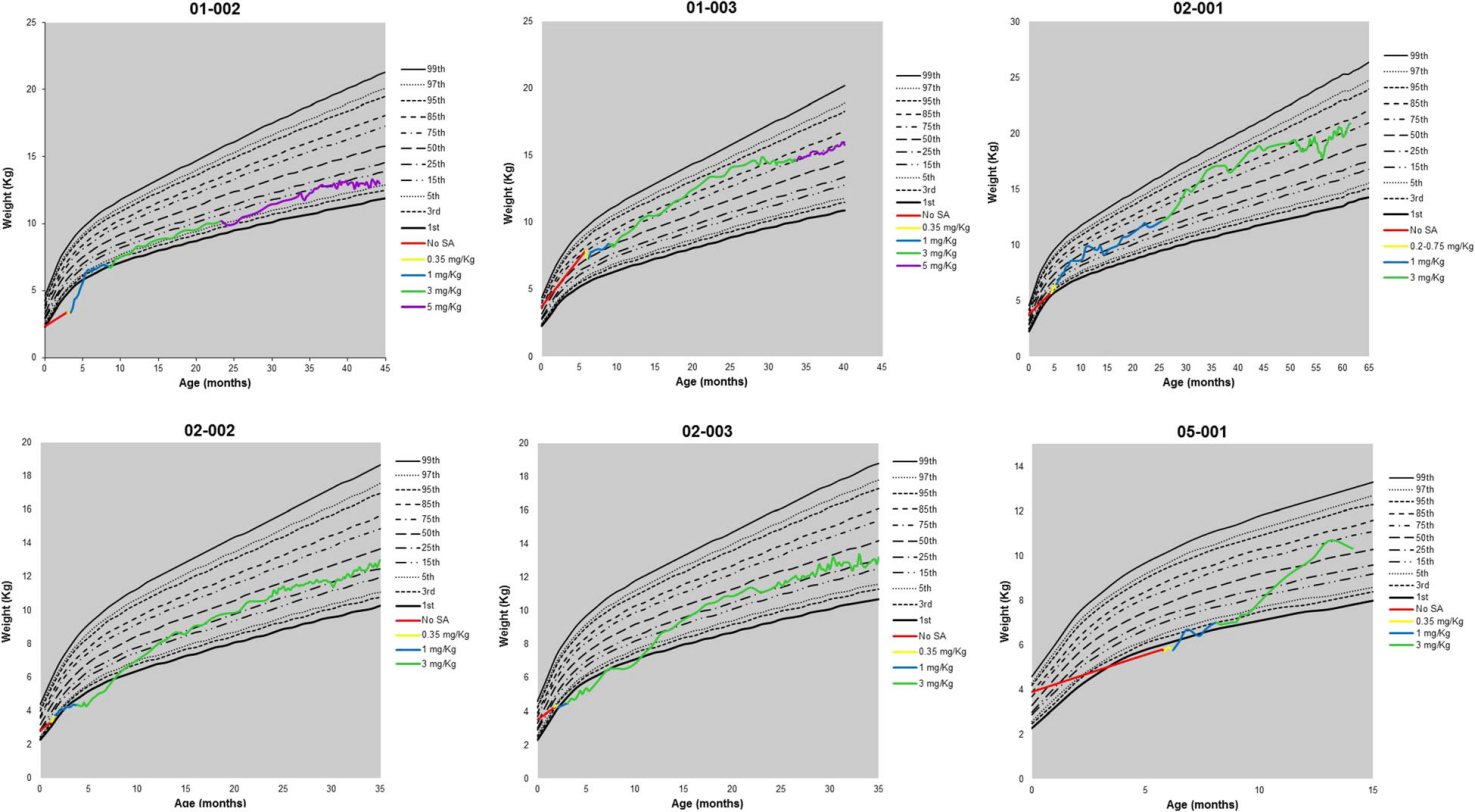
Individual growth curves over time for the 6 patients who met the primary endpoint (12-month survival). ADA = time at anti-drug antibody detection. *Due to the window of time associated with scheduled assessments, no weight was collected at 12 months for patient 05–001; therefore, the next available weight data point was used to graph the weight at 12 months; this patient died at age 15 months

All six patients who survived beyond week 4 and reached 12 months of age exhibited increases in WFA centile during treatment with sebelipase alfa (median baseline WFA, 3^rd^ [3.08] centile; *n* = 8) (Fig. 3). The age ≥24-month extension period analysis showed that two of the five surviving patients reached near or above the 75^th^ WFA centile, two patients reached near or above the 25^th^ WFA centile, and one patient remained stabilized near or above the 5^th^ WFA centile. As shown in Fig. 3, initial dose escalations occurred early during treatment. Three patients had additional dose escalations after 12 months of age; in one patient the dose was escalated from 1 mg/kg to 3 mg/kg and in two patients the doses were escalated from 3 mg/kg to 5 mg/kg. WFA centile also stabilized; at the last available assessment among the five ongoing patients, median WFA was in the 32^nd^ (31.8) centile.

At baseline, median LFA was 1.8% (*n* = 8). Post-baseline LFA data were available for six patients surviving beyond week 4; LFA fluctuated but showed an overall improvement in three patients (a decrease and return to baseline level in two patients and a decrease in one patient). The proportion of patients who met criteria for undernutrition in the primary analysis set decreased with treatment (Additional file 1: Table S4, Proportion of patients meeting criteria for undernutrition in the primary analysis [patients who survived to age 12 months]).

**Fig. 4:**
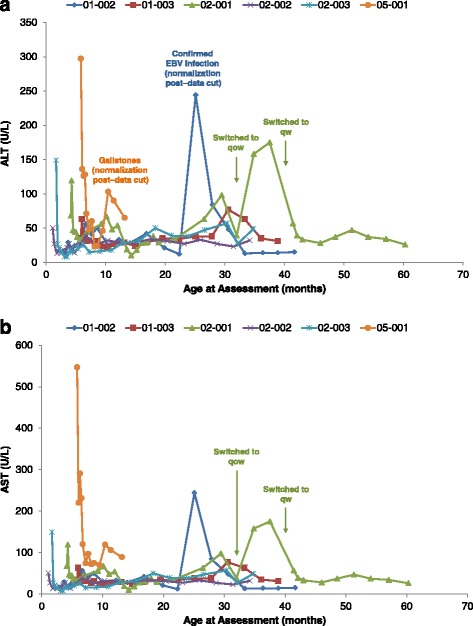
Changes in serum transaminases. Individual patient results for transaminases over time: (**a**) alanine aminotransferase (ALT) and (**b**) aspartate aminotransferase (AST). EBV = Epstein-Barr virus; qow = every-other-week dosing. To convert to SI units (μkat/L), multiply by 0.0167

Rapid decreases were noted in ALT, AST, and total bilirubin levels with sebelipase alfa treatment and these effects were seen with the initial dose of 0.35 mg/kg (Fig. 4; Additional file 1: Table S3). Normalization of transaminase levels was achieved for four of the six patients with elevated baseline AST and all four patients with elevated baseline ALT, with normal levels achieved between weeks 1 and 5. GGT levels decreased markedly over the first 3 months of treatment and stabilized thereafter, with median reductions from baseline of 40.0 U/L (0.67 μkat/L) by week 12 (*n* = 5). As of the cut-off date for the analysis at ≥24 months of age, ALT had decreased from baseline by as much as 67% and was below the upper limit of normal in four of the five patients; AST had decreased by as much as 67% (Additional file 1: Table S5). Biochemical improvements were accompanied by improvements in clinical symptoms of diarrhea, vomiting, and abdominal distension, with reductions in liver and spleen size assessed by physical examination and/or abdominal ultrasound.

Hemoglobin levels were abnormal at baseline (*n* = 9; median 93.0 g/L; range 1.4–103.0 g/L; reported as low in six patients). Five patients achieved TFHN of at least 4 weeks during treatment before the primary analysis data cut-off date. Two patients achieved TFHN maintenance (transfusion-free at week 6 and hemoglobin levels below the age-adjusted lower limit of normal at week 8 and continuing for ≥13 weeks). At the age ≥24 months analysis cut-off date, hemoglobin had increased from baseline in four of the five ongoing patients and was unchanged from baseline in the remaining patient (Additional file 1: Table S5). None of the five ongoing patients required blood transfusion since age 1 year, 11 months. At the primary analysis cut-off date, requirements for nutritional support were reduced (eliminated for two patients), and at the age ≥24 months analysis, no patients were receiving parenteral nutrition; four were receiving medium-chain triglyceride formula and a reduced-fat diet, and one was receiving an unrestricted diet. Albumin had increased from baseline in three of the five ongoing patients, and slightly decreased in one patient (Additional file 1: Table S5). Median baseline platelet count was 173.0 × 10^9^/L (*n* = 9; range 2.6–563.0 × 10^9^/L; reported as low in four patients). Platelet counts demonstrated increasing trends from baseline. Serum ferritin levels were elevated at baseline for six of the seven patients with available data; the median value was 586 μg/L (range 253–48,740 μg/L). Rapid and marked decreases in serum ferritin values were observed following initiation of treatment with sebelipase alfa. By week 1, median levels changed by −122 μg/L (range −6934 to −72 μg/L; *n* = 4) and by week 6 levels changed by −269 μg/L (range −11,171 to −215 μg/L; *n* = 3). At last reading, ferritin values for the five surviving patients were all within normal limits (range 38 to 90 μg/L). Hypertriglyceridemia was observed in four of the six patients with measurements at baseline. At the primary analysis, all four patients had decreases in serum triglycerides following initiation of sebelipase alfa and had achieved a normal serum triglyceride level. High-density lipoprotein cholesterol levels were low at baseline or the first available assessment in all six surviving patients, and increased in five of these patients during treatment. Low-density lipoprotein cholesterol levels decreased in five of the six surviving patients during treatment, and normalized in the two patients who had an elevated low-density lipoprotein cholesterol level at baseline or the first available assessment.

### Developmental milestones

Denver II Developmental Screening Test data were available at baseline for only three patients, due to a requirement that patients had to be at least 1 month of age on the date of the assessment and be sufficiently responsive to be testable for each skill area of the test. At week 24 (*n* = 5), fine motor–adaptive, language, and personal-social skills were normal in four patients and suspect in one patient. Gross motor skills were classified as normal in two patients and suspect in three patients. Data at later time points were consistent with those from week 24 in that development was found to be mostly normal, with no patient untestable for any skill area. At the age ≥24 months analysis, four of the five ongoing patients tested normal on the Denver II Developmental Screening Test assessment; one patient was assessed as normal in three of four categories but classified as suspect in language. The oldest patient in the study entered preschool at age 3 and has been attending school without any reported difficulties compared with his peers.

### Safety

Adverse events in the overall population (primary efficacy set) are summarized in Table 2; adverse events by time interval of treatment are summarized in Table 3. Reported adverse events were mainly attributable to complications and comorbidities associated with LAL deficiency. Treatment-emergent adverse events (defined in Additional file 1, eDefinition of treatment-emergent adverse events) that were reported for two or more patients are shown in Additional file 1: Table S6; 94% of the treatment-emergent adverse events reported throughout the primary treatment and extension periods were considered mild to moderate in severity. For the primary efficacy analysis, moderate to severe IARs occurred in three patients in association with 47 of the 462 infusions administered. As of January 26, 2016 a majority of the 54 IARs were mild or moderate (eg, fever, vomiting). Four treatment-related serious adverse events, which were IARs, occurred in a single patient (pyrexia, pallor, chills, tachycardia) in association with the same infusion. Three of these IARs were considered severe. The patient was hospitalized overnight and received intravenous antibiotics for possible line bacteremia; all culture results were negative. A total of 54 IARs occurred in five patients over the course of their treatment. All other IARs (Additional file 1: Table S7 for a summary of IARs) were successfully managed by modifications to infusion rates and treatment with antipyretics, antihistamines, and anti-inflammatories. None of the IARs was consistent with anaphylaxis, and none resulted in discontinuation of study treatment. Four patients developed ADAs during treatment with sebelipase alfa, only one of whom remained ADA-positive through the primary data cut-off date. Two of the four ADA-positive patients tested positive for neutralizing antibodies. In one of these patients the sebelipase alfa dose was escalated to 5 mg/kg because of concerns about the rate of weight gain.

**Table 2:** Summary of adverse events

Event, *n* (%)	Overall (*N* = 9)
Any TEAE	9 (100)
Any related TEAE	6 (67)
Any IAR	5 (56)
Any serious TEAE	9 (100)
Any related serious TEAE	1 (11)
Dose modification due to a TEAE^*^	7 (78)
Discontinuation due to a TEAE	0
Death	4 (44)
Death related to treatment	0

*IAR* infusion-associated reaction, *TEAE* treatment-emergent adverse event

^*^All dose modifications were dose escalations based on inadequate clinical response. These symptoms of inadequate clinical response were also captured as adverse events (see Additional file 1)

**Table 3:** Summary of adverse events by treatment time interval

	Treatment time interval		
Event, *n* (%)	0–3 months^*^(*n* = 9)	>3–6 months^*^(*n* = 6)	>6–12 months^*^(*n* = 6)
Any TEAE	8 (89)	5 (83)	6 (100)
Any related TEAE	4 (44)	1 (17)	3 (50)
Any IAR	3 (33)	2 (33)	3 (50)
Any serious TEAE	7 (78)	4 (67)	4 (67)
Any related serious TEAE	1 (11)	0	0
Dose modification due to a TEAE^†^	3 (33)	1 (17)	1 (17)
Discontinuation due to a TEAE	0	0	0
Death	3 (33)	0	1 (17)
Death related to treatment	0	0	0

*IAR* infusion-associated reaction, *TEAE* treatment-emergent adverse event

^*^Non-serious adverse event data were unavailable for one patient from week 0 to week 39 (~month 9)

^†^All dose modifications were dose escalations based on inadequate clinical response. These symptoms of inadequate clinical response were also captured as adverse events (see Additional file 1)

## Discussion

LAL deficiency presenting in infancy is a medical emergency with rapid disease progression and early death. Delayed diagnosis or misdiagnosis is common, as the disease is under-recognized as a cause of marked failure to thrive and rapidly progressive liver failure in infants and currently is not included in newborn screening. Historical attempts at treatment including HSCT have been almost uniformly unsuccessful [7]. Sebelipase alfa was recently approved by the US Food and Drug Administration and the European Commission for long-term ERT in patients of all ages with LAL deficiency [25]. The current report describes the first experience of sebelipase alfa use in infants with rapidly progressive LAL deficiency. In this study we found that enzyme replacement with sebelipase alfa was effective, as reflected in the outcome of survival, with six of nine patients surviving through to the primary endpoint at 12 months of age; this is in marked contrast to outcomes in a matched historical control group of untreated patients, in which no patient survived to 12 months of age (data on file, Alexion Pharmaceuticals, Inc.) [7]. The historical control group is from a natural history study intended to characterize LAL deficiency and disease progression, and to inform future clinical trial design. Inherent variability of the dataset was consistent with the small number of patients from diverse geographical areas who were diagnosed over a broad period of time. Nevertheless, these data provided valuable information on commonalities in the presentation and disease course of LAL deficiency that were confirmed by the findings of the present study.

The clinical presentation and laboratory abnormalities were consistent with those reported in the literature, with early onset and prominent failure to thrive and severe hepatic disease as evidenced by massive hepatomegaly, elevation of transaminases, hyperbilirubinemia, coagulopathy, and hypoalbuminemia [26, 27]. Adrenal calcification, which was observed in all patients in this study, is not always seen in infantile-onset LAL deficiency [7]. The abnormalities in serum triglyceride, low-density lipoprotein cholesterol, and high-density lipoprotein cholesterol levels observed for all six patients with baseline data suggest that rapidly progressive, early-onset LAL deficiency in infants is not associated with the typical dyslipidemia pattern seen in children and adults with later-onset LAL deficiency. This lipid profile in infants may be due to fat malabsorption and the resulting severely poor nutritional status of the patients. Evidence of macrophage activation was seen in some patients including cytopenias and elevated ferritin levels, likely secondary to lipid accumulation in macrophages. LAL deficiency is not typically considered in the differential diagnosis of hemophagocytic lymphohistiocytosis, which has resulted in the presumptive diagnosis and treatment for familial hemophagocytic lymphohistiocytosis in some cases [28, 29].

In addition to improved survival compared to untreated patients, survival in sebelipase alfa–treated patients was also prolonged relative to transplanted patients in the historical control group (data on file, Alexion Pharmaceuticals, Inc.) [7]. The improvements in survival with sebelipase alfa were accompanied by clinically meaningful improvements in growth, liver function, and hematological parameters and evidence of normal development. Five of the six patients who survived to 12 months of age continue with ongoing sebelipase alfa treatment. These patients have transitioned from being hospitalized in a high-dependency setting to life at home with outpatient infusions; elimination of requirements for transfusions was observed in all patients who survived to 12 months of age. It is imperative that nutritional needs be closely monitored, and frequent adjustments were made to meet the nutritional needs of each patient during treatment with sebelipase alfa. In general, all surviving patients have experienced a reduction in the need for nutritional support and none are currently receiving parenteral support. Initiation of treatment with sebelipase alfa at doses equal to or greater than 0.35 mg/kg produced rapid stabilization and/or improvements in growth and other disease-related abnormalities including markers of liver injury; this was consistent with preclinical results in an animal model of LAL deficiency (Quinn AG, et al. Abstract presented at the 2010 Annual Meeting of the American Society of Human Genetics, Washington, DC). The rapid decreases in serum transaminases observed for patients in this study are consistent with findings from a previous phase 1/2 study of sebelipase alfa in adults with LAL deficiency (LAL-CL01/CL04) [18, 30]. Five of the six patients who survived to age 12 months had their weekly dose escalated to 3.0 mg/kg after a variable period receiving 1 mg/kg, and one patient (01–002) underwent a further dose escalation to 5.0 mg/kg, primarily because of concerns related to the rate of weight gain. Another patient (01–003) was also dose escalated to 5 mg/kg weekly. This patient had been progressing well on a dose of 3 mg/kg administered once weekly and was permitted to go onto an every-other-week infusion schedule. However, this was not successful and resulted in hypoalbuminemia and disturbed transaminases. The patient was restored to a 3 mg/kg once-weekly dose schedule but showed no improvement and doses were subsequently escalated to 5 mg/kg given once weekly. At the time of the data cut-off, all five of the dose-escalated patients had improvement in WFA across two or more major centiles compared with their baseline values.

The protocol was amended during the course of the study to allow for higher doses of sebelipase alfa than were originally planned. No human studies had been conducted and there was no human experience with sebelipase alfa in LAL deficiency prior to the initiation of the current study in 2011. Given the lack of available data, safety was the most important consideration at the time the study was initiated. Thus, a starting dose of 0.35 mg/kg given once weekly was deemed to be the minimally effective dose, and it was anticipated that doses of up to 3 mg/kg would be required based on data obtained from preclinical studies of sebelipase alfa in animal models of LAL deficiency. Some patients developed antibodies to sebelipase alfa during the study, which could potentially neutralize the efficacy of the study drug. As a result of these observations, further dose escalations to 5 mg/kg per week were allowed at the discretion of the study investigators. Although improvements in a number of parameters were seen with the initial 0.35 mg/kg dose, given the rapid disease progression in infants and potential for development of ADAs, further investigation of higher sebelipase alfa doses may be warranted. Other limitations of this study are the small sample size, and the requirement to use a historical control group for the assessment of efficacy given the rapid and fatal course of the untreated disease.

Sebelipase alfa was generally well tolerated. Although IARs were seen in all patients, they were successfully managed with approaches commonly used with other ERTs. Two of the five currently ongoing patients have experienced frequent IARs; these events have been well managed by site personnel and have not impacted their treatment. The safety experience with sebelipase alfa to date appears favorable given the medical condition of infants with rapidly progressive disease.

## Conclusions

We describe the first experience with sebelipase alfa in infants presenting with LAL deficiency. The observed beneficial effects include survival well beyond the usual life expectancy in affected infants with improvements in growth, liver injury and function, reductions in hepatosplenomegaly, amelioration of cytopenias, and attainment of developmental milestones. Given the historical morbidity and mortality in infants with LAL deficiency, including rare patients treated by HSCT, early treatment with sebelipase alfa represents an important advance in the management of these patients. The rapidity of disease progression and its impact on the effects of enzyme replacement highlight the importance of early diagnosis of this life-threatening disease.

## Additional file

Additional file 1: Table S1. Summary of dosing, dose changes, and current clinical status (patients who survived to age ≥24 months). **Table S2.** LAL enzyme activity and *LIPA* genotypes. **Table S3.** Changes in serum transaminases, total bilirubin, and γ-glutamyltransferase (full analysis set). **Table S4.** Proportion of patients meeting criteria for undernutrition in the primary efficacy analysis (patients who survived to age 12 months). **Table S5.** Serum transaminases, hemoglobin, and albumin, most recent measurement (patients who survived to age ≥24 months). **Table S6.** Treatment-emergent adverse events (TEAEs) reported for two or more patients in the full analysis set. **Table S7.** Summary of infusion-associated reactions (IARs) in the full analysis set. (DOCX 94 kb)

## Abbreviations

ADA: Anti-drug antibody
ALT: Alanine aminotransferase
AST: Aspartate aminotransferase
ERT: Enzyme replacement therapy
GGT: γ-glutamyltransferase
HSCT: Hematopoietic stem cell transplantation
IAR: Infusion-associated reaction
LAL: Lysosomal acid lipase
LFA: Length-for-age
TFHN: Transfusion-free hemoglobin normalization
WFA: Weight-for-age
WHO: World Health Organization
