# Supplementary material for: Survival in infants treated with sebelipase Alfa for lysosomal acid lipase deficiency: an open-label, multicenter, dose-escalation study
Source: Orphanet J Rare Dis. 2017 Feb 8;12:25. doi: 10.1186/s13023-017-0587-3 (PMC5299659; doi:10.1186/s13023-017-0587-3)
Supplement: Additional file 1: Table S1. — Summary of dosing, dose changes, and current clinical status (patients who survived to age ≥24 months). Table S2. LAL enzyme activity and LIPA genotypes. Table S3. Changes in serum transaminases, total bilirubin, and γ-glutamyltransferase (full analysis set). Table S4. Proportion of patients meeting criteria for undernutrition in the primary efficacy analysis (patients who survived to age 12 months). Table S5. Serum transaminases, hemoglobin, and albumin, most recent measurement (patients who survived to age ≥24 months). Table S6. Treatment-emergent adverse events (TEAEs) reported for two or more patients in the full analysis set. Table S7. Summary of infusion-associated reactions (IARs) in the full analysis set. (DOCX 94 kb) [file 13023_2017_587_MOESM1_ESM.docx]

**ONLINE APPENDIX**

## Table of Contents

eStudy centers

eEvidence of rapidly progressive disease as inclusion criterion

eDose adjustment based on clinical response

eDefinition of infusion-associated reaction

eDefinition of treatment-emergent adverse events

eMeasurements

eDefinition of transfusion-free hemoglobin normalization

eStatistical methods and determination of sample size

eReference

eTable 1. Summary of dosing, dose changes, and current clinical status (patients who survived to age ≥24 months)

eTable 2. LAL enzyme activity and *LIPA* genotypes

eTable 3. Changes in serum transaminases and total bilirubin (full analysis set)

eTable 4. Proportion of patients meeting criteria for undernutrition in the primary efficacy analysis (patients who survived to age 12 months)

eTable 5. Serum transaminases, hemoglobin, and albumin, most recent assessment (patients who survived to age ≥24 months)

eTable 6. Treatment-emergent adverse events (TEAEs) reported for two or more patients in the full analysis set

eTable 7. Summary of infusion-associated reactions (IARs) in the full analysis set

## eStudy centers

This study was conducted in nine primary centers in the United Kingdom, United States, France, Turkey, Saudi Arabia, Taiwan, Italy, and Egypt and three qualified local medical centers in the United Kingdom, France, and Ireland.

**eEvidence of rapidly progressive disease as inclusion criterion**

A patient who did not meet the criteria for growth failure could be enrolled if the investigator had substantial concerns about the patient based on evidence of rapid disease progression requiring urgent medical intervention, including circumstances where the patient had an older sibling with documented rapidly progressing course of lysosomal acid lipase (LAL) deficiency with growth failure prior to 6 months of age. Inclusion under these circumstances required submission of a written summary of the patient’s medical status for review by the sponsor and approval by the sponsor after consultation with the study’s Safety Committee. The patient had to meet all other entry criteria.

## eDose adjustment based on clinical response

After taking into consideration any other potential causes for the observed clinical manifestations within a patient’s first 3 months of treatment (eg, missed study infusions, onset of acute cholecystitis or viral illness, or initiation of a potentially hepatotoxic concomitant medication), suboptimal clinical response was defined as meeting at least two of the following criteria:

- Failure to gain an average of 5 g/kg body weight per day and the presence of either of the following:
  - World Health Organization (WHO) weight-for-length or weight-for-height *z* score <−2
  - WHO length-for-age or height-for-age *z* score <−2
- Albumin less than 35 g/L
- Alanine aminotransferase greater than twice the upper limit of normal
- Ongoing requirement for blood and/or platelet transfusion

After a patient had completed at least 3 months of treatment, suboptimal clinical response was defined as any clinically important manifestation of LAL deficiency (on clinical examination, laboratory assessment, or imaging) that had not improved from baseline, had improved and plateaued (based on at least three assessments) but had not yet normalized, or failed to normalize within 12 months of treatment.

## eDefinition of infusion-associated reaction

The definition of an infusion-associated reaction was any adverse event that occurred during the 2-hour sebelipase alfa infusion or within 4 hours after the infusion and was assessed by the investigator as at least possibly related to sebelipase alfa, or any adverse event deemed by the investigator as consistent with a hypersensitivity reaction and related to administration of the study drug, regardless of timing relative to infusions. The study protocol provided guidance to the investigator on signs and symptoms of a potential infusion-associated reaction.

## eDefinition of treatment-emergent adverse events

## Treatment-emergent adverse events (TEAEs) were defined as adverse events that had an onset or increased in severity on or after the time of the first infusion of sebelipase alfa. Recurring TEAEs were counted only once per patient (by greatest severity and relationship to sebelipase alfa) when tabulating incidence rates, but each occurrence of the recurring TEAE was counted when tabulating events. Drug-related TEAEs were those reported by the investigator as related or possibly related to sebelipase alfa, and non–drug-related TEAEs were those reported by the investigator as unlikely to be related to, or not related to, sebelipase alfa.

## eMeasurements

Central laboratories were responsible for analysis of LAL enzyme activity and anti-drug antibodies (ADAs); all other clinical laboratory tests were performed by local laboratories and assessments of normal/abnormal results were based on age- and gender-specific normal ranges provided by the local laboratory at the time of the test. Serum antibodies that bind to recombinant human LAL ADAs were detected using an enzyme-linked immunosorbent assay (ELISA) and confirmed using a similar recombinant human LAL confirmatory ELISA. A patient was considered to be positive for ADAs at a given time point if he or she had a positive result on both the screening and confirmatory ELISAs.

**eDefinition of transfusion-free hemoglobin normalization**

Patients were considered to have achieved transfusion-free hemoglobin normalization (TFHN) if all of the following criteria were met:

- Two post-baseline measurements of hemoglobin, at least 4 weeks apart, were above the age-adjusted lower limit of normal (LLN)
- No known additional measurements of hemoglobin were below the age-adjusted LLN during the (minimum) 4-week period
- No transfusions were administered to the patient during the (minimum) 4-week period, or for 2 weeks prior to the first hemoglobin measurement in the (minimum) 4‑week period

If all three criteria were met, a patient was considered to have achieved TFHN on the date of the first hemoglobin assessment in the 4-week period. A patient was considered to have maintained TFHN if he or she was transfusion-free at week 6 and had no abnormally low hemoglobin levels (levels below the age-adjusted LLN) beginning at week 8 of the study and continuing for at least 13 weeks.

## eStatistical methods and determination of sample size

Continuous parameters were summarized as the number of patients with non-missing values, mean, SD, minimum, median, and maximum. Categorical parameters were summarized as frequencies (ie, number and percentage of patients in each category). For summaries of changes or percentage changes from baseline, baseline was defined as the last non-missing assessment prior to the first infusion of sebelipase alfa.

The proportion of patients surviving to 12 months of age was calculated, along with an exact 95% CI based on the Clopper-Pearson method. As a complementary analysis, Kaplan-Meier survival curves were generated from birth to 12 months of age and from the first infusion of sebelipase alfa to 12 months of age, as well as a Kaplan-Meier estimate and exact 95% CI for median survival past the first infusion of sebelipase alfa. To support a comparison of survival rates between treated patients in study LAL-CL03 and untreated infants with LAL deficiency in natural history study LAL-1-NH01, the proportion (exact 95% CI) of patients in study LAL-1-NH01 surviving to 12 months of age was re-analyzed using the Clopper-Pearson method, which provides more accurate confidence interval estimates under small sample sizes or extreme proportions, and Kaplan-Meier survival curves were constructed for LAL-1-NH01. Median age at death was estimated by Kaplan-Meier methodology.

The percentages of patients meeting criteria for the following dichotomous indicators of undernutrition^1^ were tabulated as follows: underweight (defined as <−2 SD from the median for weight-for-age *z* score); wasting (defined as less than −2 SD from the median weight-for-height *z* score); and stunting (defined as less than −2 SD from the median height-for-age *z* score).

The planned enrollment for this study was approximately ten patients, including at least eight patients who were no more than 8 months of age on the date of their first infusion of sebelipase alfa and therefore eligible for the primary efficacy analysis. This sample size was based on feasibility, taking into account the rarity of the disease, and was selected to facilitate comparison with a historical control group of infants who presented with growth failure due to LAL deficiency and did not receive hematopoietic stem cell transplant, liver transplant, or enzyme replacement therapy (ERT). Supportive sample size calculations demonstrated that for the primary efficacy analysis, if six of the eight planned patients were to survive to 12 months of age, the exact 95% CI for 12-month survival would be 34.91–96.81. By comparison, the 95% CI for 12-month survival in a similar patient population not receiving ERT (n=18) was 0.14–27.29, as determined from an analysis of the historical control group. Thus, the lower limit of the exact 95% CI in the current study, assuming six patients were to survive to 12 months of age (34.91), would exceed the upper limit of the 95% CI for 12-month survival in infants with growth failure due to LAL deficiency who did not receive ERT (27.29).

**eReference**

1. Tracking Progress on Child and Maternal Nutrition. New York, NY: United Nations Children's Fund; 2009.

**Additional file 1: Table S1** Summary of dosing, dose changes, and current clinical status (patients who survived to age ≥24 months)

| Patient | Week of 1^st^ infusion of 3 mg/kg | Current dose per kg | Anti-drug antibody | Reason for escalation to 3 mg/kg | Current status |
| --- | --- | --- | --- | --- | --- |
| 01-002 | 23 | 5 mg^*^ | Enz + CU | WFA plateaued + hepatomegaly | WFA improved, but remains low; liver function normal, liver non-palpable |
| 01-003 | 14 | 5 mg^†^ | Y, no NAb | WFA plateaued +  persistently high transaminases and low albumin | WFA >75%; liver function normal, low albumin |
| 02-001 | 91 | 3 mg | Y, no NAb | Mesenteric lymphadenopathy | WFA >50%, normal liver function, residual mild lymphadenopathy |
| 02-002 | 12 | 3 mg | No | Poor weight gain + LFA < −2 *z* score | WFA ~25%, normal liver function |
| 02-003 | 6 | 3 mg | Enz + CU | Poor weight gain + LFA < −2 *z* score | WFA ~25%, liver function normal |
| 05-001 | 10 | Deceased | No | Poor weight gain + LFA < −2 *z* score | Died at 1.25 years |

Current status is as of January 26, 2016 data cut-off.

CU = neutralizing antibody to cell uptake inhibition; Enz = neutralizing antibody to enzyme; LFA = length for age; NAb = neutralizing antibody; WFA = weight for age; Y = positive anti-drug antibody prior to escalation.

^*^ Escalated to 5 mg/kg once weekly at week 88 due to poor weight gain.

^†^ Escalated to 5 mg/kg once weekly at week 122 for persistently low albumin and high transaminases after being on 3 mg/kg every other week between weeks 96 and 108, and then 3 mg/kg every week between weeks 108 and 122.

**Additional file 1: Table S2** LAL enzyme activity and *LIPA* genotypes

|  | LAL enzyme activity | | *LIPA* genotype | | |
| --- | --- | --- | --- | --- | --- |
| Patient | PBMC^*^ (µmol/g/h) | DBS^†^ (nmol/punch) | Allelic mutations | Effect of mutation | Variant severity |
| 01-002 | 32 | ND | c.398delC, homozygous | Documented causative | Pathogenic |
| 01-003 | 41 | 0.004 | c.884A>G, heterozygous | Undocumented | Variant of unknown significance |
| 02-001 | 5 | ND | c.539-5C>T, heterozygous  c.482delA, heterozygous  c.538G>A, heterozygous | Intronic  Documented causative  Undocumented | Common variant  Pathogenic  Variant of unknown significance |
| 02-002 | ND | 0.007 | c.419G>C, homozygous | Undocumented | Variant of unknown significance |
| 02-003 | 57 | 0.007 | c.676-2A>G, homozygous | Documented causative | Pathogenic |
| 05-001 | 65 | 0.018 | c.350_351insCC, heterozygous  c.797G>T, heterozygous | Undocumented  Undocumented | Variant of unknown significance  Variant of unknown significance |

DBS = dried blood spot; ND = not done; PBMC = peripheral blood mononuclear cells.

^*^ The normal range is 350–2000 µmol/g/h.

^†^ The affected range is 0–0.016 nmol/punch for all results (normal range is 0.50–2.30 nmol/punch).

**Additional file 1: Table S3** Changes in serum transaminases, total bilirubin, and γ-glutamyltransferase (full analysis set)

|  | Observed value* | | | Change from baseline* | | |
| --- | --- | --- | --- | --- | --- | --- |
|  | n | Mean (SD) | Median (range) | N | Mean (SD) | Median (range) |
| ALT, U/L |  |  |  |  |  |  |
| Baseline | 9 | 130.1 (95.5) | 145.0 (16.0 to 297.0) | — | — | — |
| Week 2 | 5 | 95.0 (90.9) | 45.0 (21.0 to 241.0) | 5 | −60.0 (84.3) | −23.0 (−171.0 to 15.0) |
| Week 4 | 5 | 34.0 (22.8) | 31.0 (14.0 to 71.0) | 5 | −85.8 (93.0) | −33.0 (−226.0 to −4.0) |
| Week 6 | 5 | 27.0 (16.1) | 32.0 (8.0 to 48.0) | 5 | −92.8 (101.7) | −36.0 (−249.0 to −3.0) |
| Week 12 | 5 | 28.2 (10.6) | 27.0 (15.0 to 44.0) | 5 | −91.6 (113.6) | −24.0 (−273.0 to −4.0) |
| Week 24 | 5 | 44.2 (28.8) | 39.0 (15.0 to 90.0) | 5 | −65.2 (101.1) | −11.0 (−207.0 to 34.0) |
| Week 48 | 4 | 28.5 (0.6) | 28.5 (28.0 to 29.0) | 4 | −34.0 (59.6) | −13.5 (−121.0 to 12.0) |
| Week 60 | 4 | 34.3 (5.7) | 33.0 (29.0 to 42.0) | 4 | −28.3 (62.9) | −10.5 (−118.0 to 26.0) |
| Week 72 | 4 | 34.8 (11.9) | 34.0 (21.0 to 50.0) | 4 | −27.8 (48.3) | −8.5 (−99.0 to 5.0) |
| Week 96 | 5 | 82.0 (91.5) | 38.0 (27.0 to 244.0) | 5 | 18.4 (125.7) | −5.0 (−111.0 to 228.0) |
| Week 120 | 5 | 46.8 (14.3) | 48.0 (27.0 to 63.0) | 5 | −16.8 (50.6) | −23.0 (−92.0 to 32.0) |
| Week 144 | 5 | 60.2 (65.4) | 32.0 (14.0 to 175.0) | 5 | −3.4 (73.7) | −4.0 (−100.0 to 107.0) |
| Week 168 | 2 | 24.0 (12.7) | 24.0 (15.0 to 33.0) | 2 | −18.0 (24.0) | −18.0 (−35.0 to −1.0) |
| Week 192 | 1 | 37.0 (NA) | 37.0 (37.0 to 37.0) | 1 | −31.0 (NA) | −31.0 (−31.0 to −31.0) |
| Week 216 | 1 | 37.0 (NA) | 37.0 (37.0 to 37.0) | 1 | −31.0 (NA) | −31.0 (−31.0 to −31.0) |
| Week 240 | 1 | 26.0 (NA) | 26.0 (26.0 to 26.0) | 1 | −42.0 (NA) | −42.0 (−42.0 to −42.0) |
| AST, U/L |  |  |  |  |  |  |
| Baseline | 9 | 293.78 (256.06) | 125.00 (71.0 to 716.0) | — | — | — |
| Week 2 | 4 | 123.50 (112.63) | 77.00 (49.0 to 291.0) | 4 | −91.50 (110.00) | −42.50 (−256.0 to −25.0) |
| Week 4 | 4 | 69.75 (36.95) | 62.00 (35.0 to 120.0) | 4 | −139.5 (192.42) | −55.50 (−427.0 to −20.0) |
| Week 6 | 5 | 50.00 (19.20) | 48.00 (25.0 to 74.0) | 5 | −136.2 (188.85) | −62.00 (−473.0 to −31.0) |
| Week 12 | 5 | 50.20 (18.13) | 44.00 (33.0 to 75.0) | 5 | −136.00 (188.39) | −61.00 (−472.0 to −27.0) |
| Week 24 | 5 | 62.60 (32.89) | 56.00 (28.0 to 106.0) | 5 | −113.60 (185.84) | −57.00 (−441.0 to 15.0) |
| Week 48 | 4 | 39.00 (5.72) | 39.50 (32.0 to 45.0) | 4 | −44.50 (14.25) | −43.50 (−62.0 to −29.0) |
| Week 60 | 4 | 45.25 (9.54) | 43.00 (37.0 to 58.0) | 4 | −38.25 (18.89) | −41.50 (−57.0 to −13.0) |
| Week 72 | 3 | 54.3 (9.9) | 59.0 (43.0 to 61.0) | 3 | −32.0 (19.5) | −33.0 (−51.0 to −12.0) |
| Week 96 | 5 | 74.2 (43.8) | 47.0 (42.0 to 142.0) | 5 | −17.6 (48.2) | −30.0 (−49.0 to 67.0) |
| Week 120 | 5 | 61.6 (17.2) | 58.0 (46.0 to 90.0) | 5 | −30.2 (21.2) | −25.0 (−62.0 to −4.0) |
| Week 144 | 4 | 84.0 (86.0) | 49.5 (26.0 to 211.0) | 4 | −7.3 (62.6) | −33.0 (−49.0 to 86.0) |
| Week 168 | 1 | 55.0 (NA) | 55.0 (55.0 to 55.0) | 1 | −70.0 (NA) | −70.0 (−70.0 to −70.0) |
| Week 192 | 1 | 58.0 (NA) | 58.0 (58.0 to 58.0) | 1 | −67.0 (NA) | −67.0 (−67.0 to −67.0) |
| Week 216 | 1 | 72.0 (NA) | 72.0 (72.0 to 72.0) | 1 | −53.0 (NA) | −53.0 (−53.0 to −53.0) |
| Week 240 | 1 | 41.0 (NA) | 41.0 (41.0 to 41.0) | 1 | −84.0 (NA) | −84.0 (−84.0 to −84.0) |
| Bilirubin, µmol/L |  |  |  |  |  |  |
| Baseline | 8^†^ | 130.67 (200.61) | 28.95 (3.0 to 464.0) | — | — | — |
| Week 2 | 5 | 133.73 (261.61) | 10.00 (3.0 to 600.4) | 4 | 40.19 (78.13) | 1.71 (0.0 to 157.4) |
| Week 4 | 5 | 6.94 (4.68) | 4.00 (3.0 to 13.7) | 4 | −9.30 (16.66) | −1.50 (−34.2 to 0.0) |
| Week 6 | 5 | 4.97 (2.89) | 4.00 (2.0 to 9.0) | 4 | −11.26 (20.00) | −2.00 (−41.00 to 0.0) |
| Week 12 | 4 | 4.78 (3.78) | 3.57 (2.0 to 10.0) | 3 | −14.59 (24.40) | −1.00 (−42.8 to 0.0) |
| Week 24 | 5 | 2.83 (1.36) | 2.00 (2.0 to 5.1) | 4 | −12.69 (20.09) | −3.50 (−42.8 to −1.0) |
| Week 48 | 4 | 2.25 (0.50) | 2.00 (2.0 to 3.0) | 3 | −2.67 (2.52) | −3.00 (−5.0 to 0.0) |
| Week 60 | 4 | 2.50 (0.58) | 2.50 (2.0 to 3.0) | 3 | −2.33 (2.08) | −3.00 (−4.0 to 0.0) |
| GGT, U/L |  |  |  |  |  |  |
| Baseline | 8^†^ | 179.75 (335.0) | 46.50 (14.0 to 1000.0) | — | — | — |
| Week 2 | 3 | 290.67 (349.94) | 138.00 (43.0 to 691.0) | 3 | −133.67 (163.64) | −107.00 (−309.0 to 15.0) |
| Week 4 | 5 | 97.00 (108.9) | 41.0 (14.0 to 268.0) | 5 | −169.80 (319.87) | −6.00 (−732.0 to 20.0) |
| Week 6 | 5 | 73.40 (75.34) | 73.4 (15.0 to 194.0) | 5 | −193.40 (346.29) | −23.00 (−806.0 to 1.0) |
| Week 12 | 5 | 48.40 (51.30) | 14.0 (10.0 to 122.0) | 5 | −218.40 (372.20) | −40.00 (−878.0 to 0.0) |
| Week 24 | 5 | 69.60 (125.47) | 15.0 (10.0 to 294.0) | 5 | −176.20 (301.55) | −30.0 (−706.0 to −2.0) |
| Week 48 | 3 | 11.67 (3.06) | 11.0 (9.0 to 15.0) | 3 | −58.67 (70.87) | −32.0 (−139.0 to −5.0) |
| Week 60 | 3 | 11.67 (2.31) | 13. 0 (9.0 to 13.0) | 3 | −58.67 (69.37) | −34.0 (−137.0 to −5.0) |

ALT = alanine aminotransferase; AST = aspartate aminotransferase; GGT = γ-glutamyltransferase; NA = not applicable.

^*^ To convert ALT, AST, and GGT to SI units (μkat/L), multiply numbers by 0.0167.

^†^ Data were available for 8 patients at baseline.

**Additional file 1: Table S4** Proportion of patients meeting criteria for undernutrition in the primary efficacy analysis (patients who survived to age 12 months)

|  | Time point | | | | | |
| --- | --- | --- | --- | --- | --- | --- |
|  | Baseline | Week 2 | Week 4 | Week 12 | Week 24 | Week 48 |
| Stunting |  |  |  |  |  |  |
| Patients meeting criteria/evaluated, n/N^*^ | 4/8 | 3/7 | 3/6 | 1/6 | 2/5 | 1/4 |
| Proportion meeting criteria | 50% | 43% | 50% | 17% | 40% | 25% |
| Wasting |  |  |  |  |  |  |
| Patients meeting criteria/evaluated, n/N | 2/8 | 2/7 | 0/6 | 1/6 | 1/5 | 0/4 |
| Proportion meeting criteria | 25% | 29% | 0% | 17% | 20% | 0% |
| Underweight |  |  |  |  |  |  |
| Patients meeting criteria/evaluated, n/N | 2/8 | 3/7 | 2/6 | 3/6 | 3/5 | 0/4 |
| Proportion meeting criteria | 25% | 43% | 33% | 50% | 60% | 0% |
| No stunting or wasting and not underweight |  |  |  |  |  |  |
| Patients meeting criteria/evaluated, n/N | 3/8 | 3/7 | 3/6 | 3/6 | 2/5 | 3/4 |
| Proportion meeting criteria | 38% | 43% | 50% | 50% | 40% | 75% |

^*^ Data were available for 8 patients at baseline.

**Additional file 1: Table S5** Serum transaminases, hemoglobin, and albumin, most recent measurement (patients who survived to age ≥24 months)

| Parameter | 01-002 | 01-003 | 02-001 | 02-002 | 02-003 |
| --- | --- | --- | --- | --- | --- |
| ALT, percent change from baseline | −44% | −9% | −62% | −36% | −67% |
| ALT, × ULN | 0.2 | 0.7 | 0.65 | 0.4 | 1.2 |
| AST, percent change from baseline | −47% | −65% | −67% | −48% | −34% |
| Hemoglobin, percent change from baseline | 7% | 75% | 21% | 17% | No change |
| Albumin, percent change from baseline | 63% | 13% | −5% | 9% | No change |

ALT = alanine aminotransferase; AST = aspartate aminotransferase; ULN = upper limit of normal.

## Additional file 1: Table S6 Treatment-emergent adverse events (TEAEs) reported for two or more patients in the full analysis set

|  | All treated patients (N=9) | |
| --- | --- | --- |
| System/organ/class, preferred term | Events, no. | Patients, n (%) |
| Any TEAE | 461 | 9 (100) |
| Gastrointestinal disorders | 93 | 8 (89) |
| Diarrhea | 39 | 6 (67) |
| Vomiting | 32 | 6 (67) |
| Teething | 3 | 2 (22) |
| Gastroesophageal reflux disease | 2 | 2 (22) |
| Metabolism and nutrition disorders | 29 | 6 (67) |
| Decreased appetite | 5 | 2 (22) |
| Dehydration | 3 | 2 (22) |
| Vitamin D deficiency | 3 | 2 (22) |
| Vitamin K deficiency | 3 | 2 (22) |
| Vitamin E deficiency | 2 | 2 (22) |
| Metabolic acidosis | 2 | 2 (22) |
| Skin and subcutaneous tissue disorders | 35 | 6 (67) |
| Rash | 9 | 2 (22) |
| Urticaria | 8 | 3 (33) |
| Dermatitis, diaper | 7 | 4 (44) |
| Eczema | 3 | 3 (33) |
| Erythema | 2 | 2 (22) |
| Infections and infestations | 101 | 6 (67) |
| Rhinitis | 20 | 5 (56) |
| Nasopharyngitis | 8 | 5 (56) |
| Gastroenteritis | 7 | 2 (22) |
| Catheter-site infection | 6 | 3 (33) |
| Device-related infection | 6 | 3 (33) |
| Pharyngitis | 5 | 3 (33) |
| Ear infection, viral | 3 | 2 (22) |
| Viral infection | 3 | 2 (22) |
| Bronchiolitis | 2 | 2 (22) |
| Upper respiratory tract infection | 2 | 2 (22) |
| Varicella | 2 | 2 (22) |
| Ear infection | 2 | 2 (22) |
| Hand-foot-and-mouth disease | 2 | 2 (22) |
| Urinary tract infection | 2 | 2 (22) |
| General disorders and administration-site conditions | 82 | 6 (67) |
| Pyrexia | 57 | 5 (56) |
| Hyperthermia | 3 | 2 (22) |
| Irritability | 2 | 2 (22) |
| Blood and lymphatic system disorders | 10 | 6 (67) |
| Anemia | 5 | 4 (44) |
| Iron deficiency anemia | 2 | 2 (22) |
| Lymphadenopathy | 2 | 2 (22) |
| Cardiac disorders | 10 | 5 (56) |
| Tachycardia | 6 | 2 (22) |
| Bradycardia | 2 | 2 (22) |
| Respiratory, thoracic, and mediastinal disorders | 33 | 6 (67) |
| Cough | 20 | 4 (44) |
| Rhinorrhea | 4 | 2 (22) |
| Vascular disorders | 7 | 4 (44) |
| Pallor | 2 | 2 (22) |
| Congenital, familial, and genetic disorders | 3 | 3 (33) |
| Hydrocele | 2 | 2 (22) |
| Ear and labyrinth disorders | 5 | 2 (22) |
| Ear pain | 4 | 2 (22) |
| Eye disorders | 3 | 3 (33) |
| Conjunctivitis | 2 | 2 (22) |

**eTable 7** Summary of infusion-associated reactions (IARs) in the full analysis set

|  | All treated patients (N=9) | |
| --- | --- | --- |
| System/organ/class, preferred term | Events, no. | Patients, n (%) |
| Any IAR | 54 | 5 (56) |
| Cardiac disorders | 6 | 2 (22) |
| Tachycardia | 6 | 2 (22) |
| Gastrointestinal disorders | 7 | 4 (44) |
| Vomiting | 5 | 3 (33) |
| Diarrhea | 1 | 1 (11) |
| Retching | 1 | 1 (11) |
| General disorders and administration-site conditions | 28 | 3 (33) |
| Pyrexia | 19 | 3 (33) |
| Chills | 4 | 1 (11) |
| Hyperthermia | 2 | 1 (11) |
| Extravasation | 1 | 1 (11) |
| Infusion-site edema | 1 | 1 (11) |
| Irritability | 1 | 1 (11) |
| Investigations | 2 | 2 (22) |
| Body temperature increased | 1 | 1 (11) |
| Oxygen saturation decreased | 1 | 1 (11) |
| Nervous system disorders | 1 | 1 (11) |
| Hypotonia | 1 | 1 (11) |
| Psychiatric disorders | 1 | 1 (11) |
| Agitation | 1 | 1 (11) |
| Respiratory, thoracic, and mediastinal disorders | 1 | 1 (11) |
| Cough | 1 | 1 (11) |
| Skin and subcutaneous tissue disorders | 5 | 3 (33) |
| Urticaria | 4 | 3 (33) |
| Pruritus | 1 | 1 (11) |
| Vascular disorders | 3 | 2 (22) |
| Pallor | 2 | 2 (22) |
| Hypertension | 1 | 1 (11) |
